# Complementing Human Behavior Assessment by Leveraging Personal Ubiquitous Devices and Social Links: An Evaluation of the Peer-Ceived Momentary Assessment Method

**DOI:** 10.2196/15947

**Published:** 2020-08-07

**Authors:** Allan Berrocal, Waldo Concepcion, Stefano De Dominicis, Katarzyna Wac

**Affiliations:** 1 Quality of Life Technologies Lab Department of Computer Science University of Geneva Carouge Switzerland; 2 Division Of MultiOrgan Transplantation Stanford University Medical Center Stanford University Palo Alto, CA United States; 3 Department of Nutrition, Exercise and Sports University of Copenhagen Copenhagen Denmark; 4 Quality of Life Technologies Lab Department of Computer Science University of Copenhagen Copenhagen Denmark

**Keywords:** peer-ceived momentary assessment, PeerMA, ecological momentary assessment, EMA, human state assessment, behavior modeling, human-smartphone interaction, digital health, well-being, mobile phone

## Abstract

**Background:**

Ecological momentary assessment (EMA) enables individuals to self-report their subjective momentary physical and emotional states. However, certain conditions, including routine observable behaviors (eg, moods, medication adherence) as well as behaviors that may suggest declines in physical or mental health (eg, memory losses, compulsive disorders) cannot be easily and reliably measured via self-reports.

**Objective:**

This study aims to examine a method complementary to EMA, denoted as *peer-ceived momentary assessment* (PeerMA), which enables the involvement of peers (eg, family members, friends) to report their perception of the individual’s subjective physical and emotional states. In this paper, we aim to report the feasibility results and identified human factors influencing the acceptance and reliability of the PeerMA

**Methods:**

We conducted two studies of 4 weeks each, collecting self-reports from 20 participants about their stress, fatigue, anxiety, and well-being, in addition to collecting peer-reported perceptions from 27 of their peers.

**Results:**

Preliminary results showed that some of the peers reported daily assessments for stress, fatigue, anxiety, and well-being statistically equal to those reported by the participant. We also showed how pairing assessments of participants and peers in time enables a qualitative and quantitative exploration of unique research questions not possible with EMA-only based assessments. We reported on the usability and implementation aspects based on the participants’ experience to guide the use of the PeerMA to complement the information obtained via self-reports for observable behaviors and physical and emotional states among healthy individuals.

**Conclusions:**

It is possible to leverage the PeerMA method as a complement to EMA to assess constructs that fall in the realm of observable behaviors and states in healthy individuals.

## Introduction

### Background

The ecological momentary assessment (EMA) [1] method—a form of Experience Sampling Method (ESM) [2,3]—is used to collect a person’s momentary self-assessment of a particular outcome of interest (eg, mood, pain). This method has advantages such as easy interpretability, ecological validity and information richness (as the assessment comes directly from the person), self-motivation to report, and practicality [4]. This is especially useful when the objective ground truth is not obtainable; researchers thus interpret self-assessments as the ground truth of a state (eg, anxiety) [5]. This practice has disadvantages in scenarios where the self-assessment imposes an unwanted burden (eg, annoyance), the self-assessment poses a risk of reactivity [6] (eg, questions about anxiety), the person cannot answer objectively (eg, because of mental disorders), or the person chooses to answer untruthfully (eg, answers with high social desirability, always agreeing or disagreeing regardless of the question, or always picking an extreme or random response) [4].

In clinical settings, *proxies* and *observers* are often involved to inform about a patient’s condition when the patient cannot express himself or herself objectively (eg, children or patients with dementia) [7] or has limited ability to participate [8]. Outside of clinical settings, researchers have shown the value of *observers* or *peers* in identifying sources of chronic stress experienced by an individual [9] and for personality assessment [5], especially in studies where the assessment is taken only once. However, there is a lack of information regarding whether an individual’s peers (defined as close, trusted friends or family members [10]) can serve as valuable sources of information about the states and observable behaviors of the individual, potentially facilitating the early detection of certain states, including mental disorders occurring in the daily life of healthy individuals.

Consider an illustrative case of Bob in the early stages of developing an obsessive-compulsive disorder (OCD) [11] that makes him tighten his shoes irrationally. It is inherently hard for Bob to realize that his conduct is socially awkward and unsafe in locations such as stairways or busy sidewalks. Fortunately, because the behavior is observable, his wife Alice will be able to detect the early symptoms and motivate him to go for a medical check. Additionally, on the therapeutic side, Alice’s daily reports could be valuable in providing evidence that ultimately aids in his treatment. Aside from the diagnosis, peers can also be allies for an individual recovering from an addiction (eg, eating disorders, smoking, gambling). In this case, peer assessments could help prevent relapses and the associated negative consequences.

Inspired by the principles of EMA, we evaluated the peer-ceived momentary assessment (PeerMA) method, previously defined by Berrocal and Wac [10]. PeerMA is a form of EMA completed by a designated peer of an individual during the same time observation window when the individual is prompted for an EMA. Peers are asked to indicate their perception of the states of the individual as well as how confident they are about the provided assessment. The PeerMA method leverages the ubiquitous availability of smartphones as a way to collect momentary, ecologically valid data in the context of a person’s life. This enables researchers to further examine how peers could become a source of information to complement the individual’s self-assessment under certain conditions [7,9,12].

We explored 2 research aims in this study: (1) to evaluate the feasibility of the PeerMA method for studying real-life phenomena in healthy populations and (2) to identify the critical operational aspects and human factors that influence the quality of the data collected and their potential scaling. Toward this end, we conducted 2 in-the-wild studies (ie, outside the laboratory) using the PeerMA method. *Study A* was conducted around the University of Geneva (UNIGE) in Switzerland during the autumn of 2018. In this study, 13 participants self-assessed their perceived levels of stress [13], fatigue [14], and anxiety [15] multiple times a day using EMA, whereas their peers leveraged the PeerMA method to express the participants’ perceptions of the same states. *Study B* was conducted with 10 participants around Stanford University in Palo Alto, United States, in the summer of 2019. However, in this study, in addition to assessing the levels of stress, fatigue, and anxiety, participants assessed their levels of well-being [16]. Similarly, peers assessed the level of stress, fatigue, anxiety, and well-being perceived by the participant via PeerMA. Both studies lasted 28 days.

### Related Work

Both the ESM and the EMA methods were introduced in psychology [1,2] and are often used to study human aspects such as emotional awareness [17], depression [18], happiness [19], or human virtues [20] via self-reports. These methods are increasingly being used in clinical psychology [21] to study various types of disorders, such as mood dysregulation, anxiety, substance use, or psychosis [22]. EMA has also been used in patients with chronic fatigue, acquired immunodeficiency syndrome, migraine, breast cancer, or kidney disease to assess mood and stress changes [23]. Additionally, the method was adopted in organizational research [24] as well as in computer science, particularly in the subfields of ubiquitous computing and human-computer interaction [25].

#### The Proxy, Observer, Informant, or Peer Assessments

In psychology, self-assessment and other-assessment methods (also referred to as proxy, observer, informant, or peer assessments) have been used in various research contexts. For example, Vazire [5] summarized the findings of three studies providing empirical evidence of how informant-provided assessments improve the validity of personality assessments in behavioral sciences, and how they helped researchers examine questions for which self-reports alone would be insufficient. Her findings encourage researchers to incorporate informant assessments as an additional source of information to study certain human behaviors.

Gosling et al [26] studied the differences and implications of an individual’s self-reported behavioral acts (eg, expressing agreement) versus the reports made by observers of that individual after coding and judging the recorded behaviors. The focus of this research was to understand the accuracy and bias (such as self-enhancement) related to self-reports of one’s acts. Collecting observers’ assessments to examine cases of agreement and disagreement was vital for researchers to study the psychological processes of social perception.

Balsis et al [12] studied the reliability of self-reports versus informant reports in the context of personality assessment. They used the self-report and informant-report version of the Revised NEO Personality Inventory [27] inventory (including overall health measures) and showed, in a large sample (n=1449), that informant reports had greater internal consistency than self-reports for personality assessment. Although the internal consistency of informant reports does not imply that those reports are more valid than the self-reports, in this particular study, informant reports (having low variability) were better predictors of overall health measures than the self-reports of personality, which was relevant in the context of the study.

In clinical settings, in a group of older adults (*>*60 years old), Neumann et al [8] examined the validity of proxy responses used in 24 peer-reviewed publications from 1990 to 1999. Proxies (ie, family members or caregivers) showed fairly good agreement with the subject at assessing functioning, physical health, and cognitive status. However, proxies showed less agreement with the subject, reporting slightly higher impairment in emotional well-being and functioning. Such disagreement was in fact valuable in certain cases; for example, proxies provided more negative ratings than subjects during the 6 months before a hip fracture.

Focusing more on the characteristics of observers, Watson et al [28] studied the *acquaintanceship effect* in the context of self-agreements versus other agreements in low visibility aspects such as affect traits (eg, attentiveness or serenity). They involved one-time assessments of the Positive and Negative Affect Schedule Expanded Form [29], Big Five [30], and other study-specific instruments with a sample comprising 74 married couples, 136 dating couples, and 279 friendship couples. Their analysis showed that self-other agreement was significantly higher among married couples compared with dating couples or friendship dyads. Moreover, the self-other agreement was moderate to high in several components of the scales for dating couples and friendship dyads. Their work directly highlights the value of incorporating other assessments to study certain human traits, although admitting that the reliability of the other assessments should be studied carefully in each case.

Finally, although not using any form of EMA or PeerMA as presented in this paper, other studies showed empirical evidence to support 2 assumptions underlying PeerMA. Namely, (1) people often rely on their peers and trust essential information to them [31-33] and (2) peers can play a crucial role in reporting and assisting in specific scenarios such as rehabilitation and general health [8,34-36] as well as identifying the sources of chronic stress [9].

#### Value of Technology to Complement Self-Assessments

Following the trend of *personal sensing* described by Mohr et al [37], researchers have been using passively-collected raw data from smartphones and wearables to complement self-assessments of various aspects such as mental health and educational outcomes [38], stress [39-43], depressive moods [44], and schizophrenia [45]. Harari et al [46] reviewed studies using smartphone-sensing methods to identify physical movement, social interactions, and other daily activities, which can be used as objective and automated measures of behavior. More recently, Gresham et al [47] leveraged objective data from activity monitors to predict the risk of adverse events, hospitalizations, and hazard for death in advanced cancer patients. In general, these approaches profit from the abundance of passively sensed data that are converted into informative features to create computational models (commonly using machine learning or deep learning algorithms) to ultimately make inferences about human states such as those already mentioned.

However, despite its notable value, passively sensed data from smartphone sensors do not always enable accurate modeling of the perceptions of highly subjective individuals [48], and the use of such data may pose privacy risks [49] if proper data protection measures are not in place. In addition, smartphone sensor data are likely to vary in time because of hardware or sensing platform differences [37]. There is also evidence that individuals would abandon smart devices after a brief period of use [50] for reasons such as poor fit to expectations, not perceiving any direct value from the collected data, or the perceived high maintenance (eg, battery charging), especially if these devices are not their own smartphones.

We examined informant, proxy, observer reports from the literature and observed 3 main characteristics:

1. They captured individual and observer assessments using long surveys or instruments.
2. These assessments are usually carried out infrequently. Sometimes, these are one time–only assessments or are carried out every few months or years.
3. Proxies are usually involved for patients in clinical settings (due to physical or cognitive impairments).

We researched the use of PeerMA instead by (1) specifically using short surveys, for example, single-item or few-item questionnaires capturing one variable; (2) conducting frequent assessments, from >1 per day to just a few per week or month (in the case of longitudinal studies); and (3) exploring its value by focusing on healthy populations (ie, not having been diagnosed with a disease).

Our research makes a unique contribution by exploring the use of PeerMA [10] (peer assessment) to complement the information obtained via EMA (self-assessment) and personal sensing methods and collecting otherwise hard to collect data on human behaviors. The potential implications of our research are manifold, including implications for personal health, ubiquitous technologies, and mobile human-computer interaction.

## Methods

### Approach

This section describes the experimental design of the studies. To explore research aim 1 on the feasibility of the PeerMA method to study real-life phenomena in healthy populations, we collected variables such as user retention during the study, overall agreement between the EMA and PeerMA assessments, and the experimental value of the method by enabling the study of self-assessments and observer assessments paired in time. Moreover, to explore research aim 2 on operational aspects and human factors that influence the quality of the collected data, we gathered qualitative elements such as user reflections after using the method, difficulty in using the technology, and reliability of the technologies that can influence the quality of the collected data.

As explained in this section, *study B* had minor methodological differences based on lessons learned from *study A*. The studies were not designed to be replicas of each other. Instead, both studies were part of the exploratory phase of our research, in which we did not (yet) provide interventions or treatments to either the participants or their peers.

### Tools

We implemented the PeerMA method by leveraging the *mQoL Lab* platform [51,52] of the Quality of Life Technologies Research Group (UNIGE) [53]. For the 2 studies described here, we developed and published a mobile app called *mQoL Peers* available via the Google Play Store (for Android) and the Apple Store (for iOS). The mobile apps with the *mQoL Lab* platform implemented the EMA and PeerMA methods (further explained by Berrocal et al [51]), and we configured the content and frequency of the questions that the app administers via EMA and PeerMA.

### Participant and Peer Types

To be included in this study, participants and peers had to be >18 years old and own a data-enabled smartphone with Android version 8.1+ or iOS version 7+. Table 1 shows the number of participants and peers per study and the cumulative number of types of peers. We used the following 4 types of peers based on their social proximity [28]: (1) spouse, (2) dating couples, (3) relatives, and (4) friends.

For *study A*, we recruited participants around the UNIGE campus by distributing flyers, by placing advertisements at department boards, via mQoL Living Lab email distribution lists, and by word of mouth. Overall, 6 participants had 1 peer and 7 participants had 2 peers. As compensation, participants who completed the study entered a raffle for 2 Amazon gift cards worth US $50 each.

For *study B*, we recruited participants around the Stanford University campus by distributing flyers, by placing advertisements on Craigslist and department boards, via email distribution lists, and by word of mouth. A total of 7 participants had 1 peer and 3 participants had no peers (included in Table 1 and excluded from the analysis). As compensation, participants and peers who completed the study received an Amazon gift card worth US $50 each.

### Study Design: Study Surveys, EMA, and PeerMA

This section explains the entry, ambulatory, and exit surveys from each study. These are summarized in Table 2.

**Table 1:** Study participants: type and gender distribution.

Studies		Participants, n (%)	Peers, n (%)
**Study A^a^**			
	Male	7 (54)	12 (60)
	Female	6 (46)	8 (40)
**Study B^b^**			
	Male	2 (20)	3 (43)
	Female	8 (80)	4 (57)

^a^Total number of participants is 13, and total number of peers is 20.

^b^Total number of participants is 10, and total number of peers is 7.

**Table 2:** Study design: surveys and ecological momentary assessment/peer-ceived momentary assessment content in each study.

Types of survey	Study A	Study B
Entry surveys	Study entry, GERT^a^, PSS^b^, and SDS^c^	Study entry, PSS^b^, and SDS^c^
Daily surveys: EMA^d^ and PeerMA^e^	Stress, fatigue, and anxiety; frequency: 8 times a day (9 AM-9 PM); silent push notification; expires after 40 min	Stress, fatigue, anxiety, and well-being; frequency: 3 times a day (8 AM-8 PM); silent push notification; expires after 30 min
Exit survey	Study exit	Study exit

^a^GERT: Geneva Emotion Recognition Test (0-42; higher scores reflect higher ability).

^b^PSS: Perceived Stress Scale (0-40; higher scores reflect higher perceived stress).

^c^SDS: Social Desirability Scale (0-10; higher scores reflect higher social approval concern).

^d^EMA: ecological momentary assessment.

^e^PeerMA: peer-ceived momentary assessment.

### Entry Surveys

Participants and peers initially completed the entry surveys before beginning the daily EMA/PeerMA. The study entry survey had 2 parts: (1) socioeconomic status, including gender, age range, education, marital status, and employment status and (2) open-ended questions asking participants whether they considered themselves stressed, what causes their stress, and whether they think others notice when they are stressed. For peers, open-ended questions asked whether they noticed when people around them project stress, what signs they observe in those projecting stress, how they react when someone around projects stress, and whether they get stressed or change their behavior when exposed to someone who is stressed. Peers also indicated their relationship with the participant (eg, friend, spouse) and whether they cohabit with the participant.

We employed the 42-item Geneva Emotion Recognition Test (GERT) that measures a person’s ability to recognize someone’s emotions from facial, voice, and body inputs (higher scores reflect higher ability) [54]. We used the 10-item Perceived Stress Scale (PSS) to measure self-perceived stress (higher scores reflect higher perceived stress) [55]. We used the 13-item Social Desirability Scale (SDS) to measure the degree to which a person is concerned with social approval (higher scores reflect higher social approval concern) [56].

For *study B*, the entry surveys had 2 differences. First, we asked participants (not only peers) to indicate whether they noticed when individuals around them project stress. Second, we excluded the GERT test because several participants and peers in *study A* reported technical issues with its web interface (provided as a service by the hosting agency) when completing the test.

### Ambulatory Monitoring: EMA/PeerMA and Well-Being

**Figure 1:**
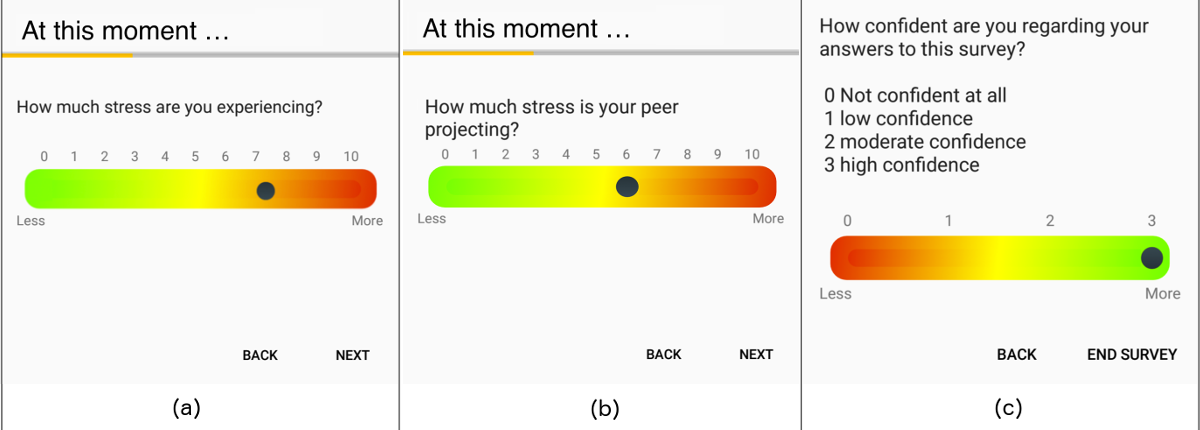
Examples of ambulatory assessment: (a) Self-assessment (ecological momentary assessment) of stress, (b) peer- assessment (peer-ceived momentary assessment) of stress, (c) confidence assessment required from peers. Assessments of fatigue, anxiety, and well-being followed the same approach.

In both studies, we used single-item questions proposed by Rosenzveig et al [57] on a visual analog scale, as shown in Figure 1. We chose stress, fatigue, and (state) anxiety because they can be studied using introquestive methods [58]. They often occur among healthy adults [59]. They compromise a person’s well-being (eg, high stress, bad sleep quality) [60] and are not trivially observable by peers, and presumably, early detection of these conditions could inform diagnosis or therapeutic decisions. Additionally, these states can vary during a day and hence are good candidates to report with EMA/PeerMA triggered through a day.

For *study A*, we used signal-contingent triggers between 9 AM and 9 PM for a total of 8 per day. They were uniformly randomized and separated by ≥45 min. Participants and peers received the EMA and PeerMA via silent push notifications (no sound and no vibration), which expired after 30 min if unanswered by the participant (to prevent questions from piling up). With EMA, we asked the following questions: “How much {stress, fatigue, anxiety} are you experiencing?“ Correspondingly, with PeerMA, we asked: “How much {stress, fatigue, anxiety} is your peer projecting?” in addition to the peer’s confidence assessment (Figure 1), which allowed peers to indicate how confident they were with each assessment. In principle, being next to the participant does not guarantee that peers can report with high confidence. Similarly, not being beside the participant or not having had a recent face-to-face contact does not preclude peers from reporting with high confidence. The exact dynamics used by the peers to make their observations are complex in nature, as already noted by Uher et al [58]; therefore, given the exploratory nature of this study, we decided to leave them for future work. The confidence assessment provided by peers can be used to inform the data analysis, for instance, to discard zero-labeled confidence assessments.

For *study B*, we used signal-contingent triggers between 8 AM and 8 PM for a total of 3 per day. They were uniformly randomized and separated by ≥2 hours. Participants and peers received the EMA and PeerMA via silent push notifications (no sound and no vibration), which also expired after 30 min if not addressed by the participant. With EMA, we asked the following questions: “How much {stress, fatigue, anxiety} are you experiencing?“ on a scale of 0 to 10 (the higher the worse), “How well are you?”—related to the overall level of well-being on a scale of 0 to 10 (the higher the better)—and an open question “If you wish, briefly describe your emotional state at this moment.“ Correspondingly, with PeerMA, we asked “How much {stress, fatigue, anxiety, well-being} is your peer projecting?” (using the same scale of 0-10), 1 open question “If you wish, briefly describe the state your peer projects at this moment,“ and the peer’s confidence assessment. In this study, the *mQoL Peers* app also had a button to let participants and peers self-trigger an assessment (EMA or PeerMA correspondingly) when they wished to do so.

Because the explicit well-being question had been adopted only in *study B*, in *study A* we computed it afterward. In *study A*, we adopted the notion of computed well-being (additional to the *reported well-being* in *study B*), representing a reduced, yet semantically aligned form of well-being as defined by Huppert [16]. We used a pragmatic definition of well-being as 1 minus the arithmetic mean of stress, fatigue, and anxiety. Empirically, a high prevalence of stress, fatigue, and anxiety reduces well-being. On the contrary, low levels of stress, fatigue, and anxiety are likely to contribute to a healthy state of well-being.

#### Exit Survey

At the end of the study, both participants and peers completed an exit survey commenting on usability aspects of the mobile device app (eg, usability, positive and negative aspects perceived). The survey also asked how participants felt about reflecting on their states during the day, whereas peers answered how they felt about reflecting on their peers’ states during the day.

### Study Protocol and Ethical Approval

At the beginning of the study, participants had a 15-min web-based or face-to-face meeting with the researcher with the following objectives: (1) explain the nature of the study, (2) hand out the informed consent, (3) train the participant to use the *mQoL Peers* app (concretely, how to invite peers, how to complete entry surveys, and how to address the push notifications), and (4) answer any questions from the participant.

During this meeting, the researcher explained to the participants that, given the nature of the study, peers had to be people with whom they had regular contact (at least daily), either face-to-face or virtually, using communication tools. We explained that peers could be spouses (significant others), close relatives (family), or friends from school or work. After the meeting, participants would complete the entry surveys, enroll their peers, and explain to them how to use the app. In these 2 studies, the researcher had no interaction with peers. After enrolling their peers and completing the entry surveys, participants pushed a button in the app to start the study and receive daily EMAs and PeerMAs. The researchers were in touch with the participants remotely to follow-up with them about these steps, if needed.

*Study A* was approved by the institutional review board of UNIGE under the protocol “Exploring the Value of Social Links and Human-Machine Collaboration in the Context of Stress Assessment,” N. CUREG.201807. *Study B* was approved by the Panel on Human Subjects in Medical Research of Stanford University under the protocol “Studying the Subjective and Objective Momentary Perception of Quality of Life in Different Contexts of Daily Life,” N.47833, Reg# 351.

## Results

### Structure

The first part, the section on *Feasibility of Using PeerMA,* presents the results directly associated with research aim 1 (feasibility of using PeerMA in real-life phenomena). Recalling from these methods, these results allowed us to observe variables such as user retention and completion rates in the study, the overall agreement between EMA and PeerMA assessments, and the experimental value of the method to study certain research questions. The first part is organized as follows: the section on *Collected Data Summary* introduces the data sets, including visualizations, to illustrate the contributions of the PeerMA method in the section on *Visual Explorations of Daily Dynamics.* Then it presents results from 3 analyses: directional accuracy in the section on *Mean Directional Accuracy of EMA/PeerMA,* correlations in the section on *EMA and PeerMA Correlations,* and statistical agreement between participants and peers assessments in the section on *EMA/PeerMA Statistical Agreement.*

The second part, the section on *Operational and Human Factors,* presents results associated with research aim 2 (operational and human factors). The second part of the section is organized as follows: the section on *Users’ Reflections about the Studies* summarizes the qualitative results related to the users’ reflections from both studies. Then, the section on *Implications of Technology Choices* briefly describes implications of the technology choices that we experienced in the studies. Finally, the section on *Suggestions from Users about Technology and Methods* shows some recommendations or observations made by participants in the study, which are worth sharing with the research community.

### Feasibility of Using PeerMA

We present the type of quantitative and qualitative data that are being obtained with PeerMA as a method and tool in the 2 observational studies, and not necessarily the strength of the results regarding stress, fatigue, anxiety, and well-being that have been explored as use cases. Nevertheless, we also present a detailed examination of the results to support the findings and observations that come along the analyses.

#### Collected Data Summary

The first part of the dataset, extracted from the entry surveys, describes the samples in each study. Table 3 shows the socioeconomic characteristics (gender, age, marital status, education, and employment) as well as the distribution of participants and peers who admit whether other people notice (or not) when they are stressed. Table 4 shows the test scores (GERT, PSS, and SDS) as well as the distribution of how stressed participants and peers considered themselves and how well they report to notice stress in others. The second part of the dataset contains information about the quantity and values obtained from the EMA and PeerMA in each study.

In *study A*, which included 13 participants and 20 peers, we collected a total of 878 person-days, 380 participant-days (mean 29, SD 1*.*7), and 498 peer-days (mean 25, SD 5*.*9). Participants received 3086 EMAs (mean 237, SD 32*.*3), and responded to a total of 2001 EMAs (mean 154, SD 62*.*2) with an average response rate of 65% (SD 24*.*2%). Peers received 3178 PeerMAs (mean 159*,* SD 49*.*0), and responded to a total of 1328 PeerMAs (mean 66*,* SD 37*.*9) with an average response rate of 44% (SD 24*.*5%).

In *study B*, which included 10 participants and 7 peers, we collected a total of 373 person-days, 187 participant-days (mean 27, SD 4*.*5), and 186 peer-days (mean 27, SD 4*.*8). Participants received in total 561 EMAs (mean 80, SD 13*.*4), and responded to a total of 445 EMAs (mean 64, SD 16*.*2) with an average response rate of 80% (SD 19*.*6%). Peers received 558 PeerMAs (mean 80, SD 14*.*5), and responded to a total of 432 PeerMAs (mean 62, SD 45*.*4) with an average response rate of 77% (SD 48%; including peer S1P1, who provided almost twice the expected number of PeerMAs).

**Table 3:** Participants’ socioeconomic characteristics by study.

Variables		Study A		Study B		
		Participants, n (%)	Peers, n (%)		Participants, n (%)	Peers, n (%)
**Gender**						
	Male	6 (46)	8 (40)		2 (29)	3 (43)
	Female	7 (54)	12 (60)		5 (71)	4 (57)
**Age (years)**						
	18-20	1 (8)	2 (10)		0 (0)	0 (0)
	21-29	9 (69)	8 (40)		3 (43)	3 (43)
	30-39	2 (15)	4 (20)		4 (57)	4 (57)
	40-49	1 (8)	6 (30)		0 (0)	0 (0)
**Marital status**						
	Single	10 (77)	12 (60)		3 (43)	3 (43)
	Married	2 (15)	5 (25)		4 (57)	4 (57)
	Other	1 (8)	3 (15)		0 (0)	0 (0)
**Highest education**						
	Undergraduate	8 (62)	11 (55)		1 (14)	3 (43)
	Graduate	5 (38)	9 (45)		6 (86)	4 (57)
**Currently employed**						
	Yes	3 (23)	9 (45)		3 (43)	6 (86)
	No	10 (77)	11 (55)		4 (57)	1 (14)
**Others notice my stress?**						
	Yes	9 (69)	15 (75)		7 (100)	6 (86)
	No	4 (31)	5 (25)		0 (0)	1 (14)

**Table 4:** Survey scores of participants and peers by study.

Instruments and roles		Study A			Study B		
		Minimum score	Maximum score	Mean (SD)	Minimum score	Maximum score	Mean (SD)
**GERT^a^** **(0-42)**							
	Participant	18	33	26 (5.2)	N/A^b^	N/A	N/A
	Peer	20	31	25 (3.8)	N/A	N/A	N/A
**PSS^c^** **(0-40)**							
	Participant	12	31	23 (5.9)	19	31	24 (4.6)
	Peer	0	36	22 (8.2)	12	28	21 (5.9)
**SDS^d^** **(0-13)**							
	Participant	3	12	7 (2.5)	0	10	5 (3.2)
	Peer	0	11	6 (3.0)	1	11	6 (3.4)
**Considered stressed (0-10)**							
	Participant	2.8	9.8	6.1 (2.4)	3.1	9.0	6.2 (2.4)
	Peer	0.3	8.7	4.5 (2.6)	1.9	7.9	5.0 (1.8)
**I notice other’s stress (0-10)**							
	Participant	N/A	N/A	N/A	4.0	8.9	5.7 (1.7)
	Peer	3.9	8.2	6.9 (1.07)	5.2	10	7.5 (1.5)

^a^GERT: Geneva Emotion Recognition Test.

^b^N/A: not applicable.

^c^PSS: Perceived Stress Scale.

^d^SDS: Social Desirability Scale.

Table 5 shows the number of days each person participated in the study and the response rate, computed as the percentage of EMAs/PeerMAs answered, from all triggered. For *study A*, *triggered* represents the number of EMAs/PeerMAs automatically triggered by the app. For *study B*, the response rate includes responses from *automatically triggered* plus *self-triggered* EMA/PeerMAs because, in *study B*, participants and peers were able to initiate reports voluntarily in addition to the automatic reports. The column “EMAs or PeerMAs Triggered“ shows the minimum number of expected responses for each person (in this case, 3 per day). Additionally, Table 5 shows the type of relationship between the peer and the subject, as indicated by the peer. In this paper, however, given the small number of participants, we did not report results by relationship type.

**Table 5:** Summary of the engagement of participants and peers.

Studies, participant ID		Peer ID	Days	Ecological momentary assessments or peer-ceived momentary assessments triggered	Response rate, %	Peer-participant relationship
**Study A**						
	S1	N/A^a^	27	220	61.8	N/A
	N/A	S1P1	27	127	87.4	3: parent
	S2	N/A	31	147	68.0	N/A
	N/A	S2P1	31	100	61.0	3: parent
	S3	N/A	29	243	97.9	N/A
	N/A	S3P1	29	220	35.5	4: friend
	S4	N/A	28	211	36.5	N/A
	N/A	S4P1	27	165	15.2	3: parent
	S5	N/A	29	265	47.9	N/A
	N/A	S5P1	27	252	23.0	3: sibling
	S6	N/A	29	243	97.5	N/A
	N/A	S6P1	22	183	61.7	2: boyfriend
	S7	N/A	29	245	70.6	N/A
	N/A	S7P1	20	122	30.3	4: friend
	N/A	S7P2	29	199	50.8	3: parent
	S8	N/A	28	250	94.0	N/A
	N/A	S8P1	26	214	32.2	4: friend
	N/A	S8P2	28	213	61.5	2: boyfriend
	S9	N/A	34	225	44.9	N/A
	N/A	S9P1	33	130	36.9	4: friend
	N/A	S9P2	33	133	45.9	3: sibling
	S10	N/A	29	267	31.1	N/A
	N/A	S10P1	30	102	41.2	2: girlfriend
	N/A	S10P2	16	152	4.6	4: friend
	S11	N/A	29	250	82.0	N/A
	N/A	S11P1	22	153	86.3	4: friend
	N/A	S11P2	17	100	50.0	3: sibling
	S12	N/A	28	252	77.4	N/A
	N/A	S12P1	26	198	13.1	3: sibling
	N/A	S12P2	26	206	50.0	4: friend
	S13	N/A	30	268	35.1	N/A
	N/A	S13P1	15	130	9.2	3: parent
	N/A	S13P2	14	79	79.7	4: friend
**Study B**						
	S1	N/A	31	93	69.9	N/A
	N/A	S1P1	31	93	172.0	4: friend
	S2	N/A	28	84	78.6	N/A
	N/A	S2P1	28	84	77.4	4: friend
	S3	N/A	28	84	108.3	N/A
	N/A	S3P1	28	84	60.7	1: spouse
	S4	N/A	28	84	69.0	N/A
	N/A	S4P1	28	84	59.5	1: spouse
	S5	N/A	28	84	50.0	N/A
	N/A	S5P1	27	81	28.4	1: spouse
	S6	N/A	17	51	96.1	N/A
	N/A	S6P1	16	48	102.1	4: friend
	S7	N/A	27	81	91.4	N/A
	N/A	S7P1	28	84	40.5	4: friend

^a^N/A: not applicable.

As noted in Table 5, some participants and peers deviated from the expected 28 days of participation in the study. Those with <28 days stopped participating in the study after informing the research team (S13P1 in *study A* and S6 and S6P1 in *study B*) or stopped participating without notification. On the other hand, those with >28 days were due to, in study A, the smartphone operating system at times cutting off the service that kept the daily counter in our mobile app in response to users’ settings of the battery-saving profile. Consequently, some dyads went beyond 28 days. Finally, in *study B,* S1 and S1P1 continued contributing assessments before noticing the study had ended (which are displayed on the home screen of the mobile app), at which time they completed the exit survey and completed the study.

Additionally, in Table 5, the number of triggered surveys in *study A* is smaller than the expected value (8 times the number of days) due to a failure in the mobile app that did not count notifications that expired after 30 min. On the other hand, when the user interrupted the completion of a survey (eg, by opening another app), upon returning to the survey, a new record was mistakenly added to the triggered list, resulting in a number larger than the expected value (8 times the number of days). Both issues were later resolved in the platform.

For each participant and peer, we normalized the EMA/PeerMA assessments to 0 to 1 based on the highest and lowest assessment given by each person. Table 6 shows the median, mean, and SD of all assessments for stress, fatigue, and anxiety in study A. The last 3 columns show the calculated value and computed well-being as defined in the *Methods* section. In Table 6, lower values in the “Median” and “Mean” columns represent more desirable states, whereas higher values represent less desirable states. Table 7 shows the dataset for *study B*. In addition to the computed well-being, this study shows the actual *reported well-being*, including the median, mean, and SD.

**Table 6:** Study A: Summary of the ecological momentary assessment and peer-ceived momentary assessment values. Each row shows the median, mean, and SD for the corresponding participant or peer calculated from all the assessments issued by that person.

Participant ID	Peer ID	Stress (0-1)		Fatigue (0-1)		Anxiety (0-1)		Computed well-being (0-1)	
		Median	Mean (SD)	Median	Mean (SD)	Median	Mean (SD)	Median	Mean (SD)
S1	N/A^a^	0.35	0.41 (0.27)	0.22	0.27 (0.23)	0.30	0.37 (0.27)	0.69	0.65 (0.21)
N/A	S1P1	0.70	0.62 (0.27)	0.62	0.65 (0.27)	0.70	0.57 (0.29)	0.35	0.39 (0.27)
S2	N/A	0.17	0.28 (0.29)	0.29	0.31 (0.26)	0.19	0.31 (0.33)	0.65	0.70 (0.23)
N/A	S2P1	0.72	0.62 (0.30)	0.58	0.59 (0.28)	0.37	0.34 (0.23)	0.42	0.48 (0.23)
S3	N/A	0.61	0.51 (0.30)	0.61	0.62 (0.27)	0.37	0.39 (0.24)	0.40	0.49 (0.22)
N/A	S3P1	0.52	0.49 (0.27)	0.39	0.40 (0.21)	0.48	0.50 (0.27)	0.55	0.54 (0.22)
S4	N/A	0.18	0.26 (0.26)	0.30	0.36 (0.25)	0.35	0.43 (0.32)	0.71	0.65 (0.17)
N/A	S4P1	0.65	0.60 (0.30)	0.65	0.50 (0.36)	0.79	0.63 (0.33)	0.35	0.42 (0.28)
S5	N/A	0.11	0.23 (0.28)	0.35	0.36 (0.24)	0.00	0.10 (0.23)	0.82	0.77 (0.17)
N/A	S5P1	0.64	0.58 (0.32)	0.60	0.58 (0.33)	0.12	0.19 (0.28)	0.53	0.55 (0.27)
S6	N/A	0.37	0.44 (0.28)	0.32	0.34 (0.23)	0.40	0.43 (0.22)	0.74	0.60 (0.19)
N/A	S6P1	0.49	0.49 (0.23)	0.38	0.45 (0.32)	0.46	0.43 (0.24)	0.55	0.55 (0.24)
S7	N/A	0.13	0.17 (0.20)	0.36	0.37 (0.25)	0.12	0.15 (0.20)	0.80	0.77 (0.16)
N/A	S7P1	0.55	0.58 (0.22)	0.64	0.64 (0.25)	0.68	0.60 (0.23)	0.36	0.39 (0.22)
N/A	S7P2	0.31	0.34 (0.22)	0.65	0.57 (0.29)	0.34	0.39 (0.29)	0.60	0.57 (0.21)
S8	N/A	0.08	0.18 (0.25)	0.52	0.48 (0.25)	0.07	0.14 (0.22)	0.75	0.73 (0.18)
N/A	S8P1	0.70	0.68 (0.19)	0.62	0.65 (0.18)	0.67	0.57 (0.23)	0.34	0.37 (0.17)
N/A	S8P2	0.43	0.41 (0.25)	0.58	0.55 (0.27)	0.47	0.47 (0.25)	0.50	0.52 (0.24)
S9	N/A	0.00	0.16 (0.26)	0.38	0.43 (0.30)	0.26	0.27 (0.29)	0.74	0.71 (0.19)
N/A	S9P1	0.42	0.43 (0.31)	0.67	0.61 (0.29)	0.68	0.56 (0.25)	0.42	0.47 (0.21)
N/A	S9P2	0.50	0.55 (0.27)	0.51	0.55 (0.24)	0.45	0.58 (0.34)	0.45	0.44 (0.20)
S10	N/A	0.58	0.61 (0.32)	0.58	0.57 (0.27)	0.68	0.61 (0.28)	0.37	0.40 (0.12)
N/A	S10P1	0.36	0.38 (0.28)	0.34	0.34 (0.20)	0.34	0.44 (0.28)	0.63	0.62 (0.22)
N/A	S10P2	0.19	0.39 (0.43)	0.77	0.61 (0.39)	0.42	0.48 (0.46)	0.52	0.51 (0.35)
S11	N/A	0.48	0.52 (0.23)	0.18	0.29 (0.28)	0.41	0.42 (0.22)	0.62	0.59 (0.18)
N/A	S11P1	0.49	0.49 (0.23)	0.40	0.45 (0.31)	0.52	0.51 (0.25)	0.52	0.52 (0.24)
N/A	S11P2	0.48	0.42 (0.34)	0.63	0.57 (0.37)	0.50	0.50 (0.24)	0.52	0.50 (0.28)
S12	N/A	0.36	0.46 (0.29)	0.60	0.55 (0.27)	0.07	0.19 (0.28)	0.63	0.60 (0.17)
N/A	S12P1	0.67	0.56 (0.33)	0.77	0.65 (0.30)	0.57	0.49 (0.31)	0.31	0.43 (0.29)
N/A	S12P2	0.10	0.24 (0.32)	0.53	0.52 (0.25)	0.00	0.19 (0.29)	0.74	0.68 (0.20)
S13	N/A	0.38	0.40 (0.30)	0.03	0.16 (0.24)	0.30	0.31 (0.25)	0.74	0.71 (0.18)
N/A	S13P1	0.43	0.47 (0.36)	0.45	0.50 (0.30)	0.41	0.38 (0.33)	0.56	0.55 (0.28)
N/A	S13P2	0.51	0.55 (0.27)	0.42	0.43 (0.26)	0.61	0.62 (0.22)	0.48	0.47 (0.24)

^a^N/A: not applicable.

**Table 7:** Study B: Summary of ecological momentary assessment and peer-ceived momentary assessment values. Each row shows the median, mean, and SD for the corresponding participant or peer calculated from all the assessments issued by that person.

Participant ID	Peer ID	Stress (0-1)		Fatigue (0-1)		Anxiety (0-1)		Reported well-being (0-1)		Computed well-being (0-1)	
		Median	Mean (SD)	Median	Mean (SD)	Median	Mean (SD)	Median	Mean (SD)	Median	Mean (SD)
S1	N/A^a^	0.49	0.36 (0.55)	0.58	0.37 (0.60)	0.58	0.66 (0.32)	0.76	0.72 (0.24)	0.39	0.52 (0.30)
N/A	S1P1	0.44	0.44 (0.21)	0.50	0.47 (0.23)	0.57	0.54 (0.21)	0.41	0.41 (0.21)	0.51	0.49 (0.21)
S2	N/A	0.35	0.34 (0.29)	0.42	0.44 (0.27)	0.42	0.43 (0.25)	0.73	0.63 (0.28)	0.66	0.63 (0.28)
N/A	S2P1	0.76	0.69 (0.24)	0.59	0.57 (0.20)	0.66	0.64 (0.25)	0.61	0.56 (0.29)	0.26	0.35 (0.22)
S3	N/A	0.50	0.49 (0.27)	0.60	0.58 (0.27)	0.39	0.42 (0.23)	0.48	0.47 (0.26)	0.37	0.43 (0.28)
N/A	S3P1	0.47	0.43 (0.24)	0.58	0.53 (0.32)	0.59	0.59 (0.23)	0.79	0.76 (0.20)	0.39	0.43 (0.29)
S4	N/A	0.50	0.53 (0.25)	0.56	0.59 (0.25)	0.42	0.44 (0.31)	0.64	0.56 (0.24)	0.56	0.52 (0.25)
N/A	S4P1	0.63	0.66 (0.28)	0.75	0.67 (0.25)	0.69	0.59 (0.32)	0.65	0.62 (0.28)	0.31	0.39 (0.30)
S5	N/A	0.22	0.27 (0.26)	0.15	0.24 (0.27)	0.33	0.34 (0.33)	0.78	0.66 (0.32)	0.67	0.67 (0.27)
N/A	S5P1	0.44	0.40 (0.29)	0.31	0.41 (0.32)	0.43	0.43 (0.30)	0.63	0.54 (0.29)	0.59	0.56 (0.29)
S6	N/A	0.54	0.56 (0.27)	0.53	0.54 (0.24)	0.55	0.59 (0.29)	0.48	0.51 (0.23)	0.45	0.39 (0.26)
N/A	S6P1	0.49	0.47 (0.28)	0.51	0.50 (0.29)	0.62	0.59 (0.32)	0.55	0.49 (0.32)	0.47	0.48 (0.29)
S7	N/A	0.00	0.21 (0.33)	0.54	0.56 (0.20)	0.26	0.35 (0.20)	0.62	0.59 (0.30)	0.81	0.66 (0.27)
N/A	S7P1	0.36	0.35 (0.34)	0.55	0.56 (0.28)	0.37	0.41 (0.36)	0.60	0.53 (0.26)	0.66	0.59 (0.30)

^a^N/A: not applicable.

#### Visual Exploration of Daily Dynamics

As this study primarily focused on assessing the feasibility of the method, we started the data analysis with the least complex visualization of raw datasets. We wanted to plot the values reported by the participants and their peers and understand the magnitude of agreement/disagreement in their ratings in time. We also imputed the missing PeerMA values using a spline function of order 4.

**Figure 2:**
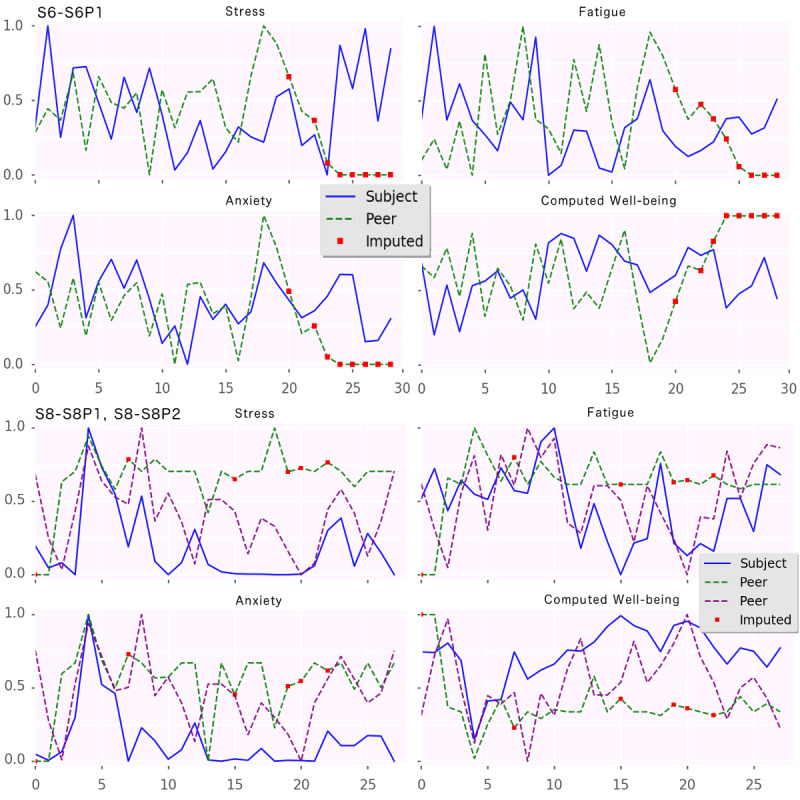
Ecological momentary assessment/peer-ceived momentary assessment. Plots from Study A. The x-axis represents days in the study, and the y-axis represents the magnitude of the normalized assessments for stress, fatigue, anxiety, and computed well-being.

To illustrate, sample plots from *study A* are presented in Figure 2. For the dyad *<*S6, S6P1*>* shown in the 4 plots on the top, despite the differences in magnitude and time shifts, there are notable similarities for states such as stress and anxiety as well as nonnegligible overlapping for fatigue. For the triad *<*S8, S8P1, S8P2*>* shown in the 4 bottom plots, there is a pattern for stress and anxiety, where S8 started to report low values although the peers continued to report higher values with periods of mutual agreement. One can see that in this triad, there seemed to be higher agreement for fatigue. Consequently, either the peers were unable to truthfully report stress and anxiety or the participant did not truthfully report stress and anxiety (intentionally or otherwise). Both are hypotheses that can be explored further with focused experiments combining EMA and PeerMA with other qualitative methods.

**Figure 3:**
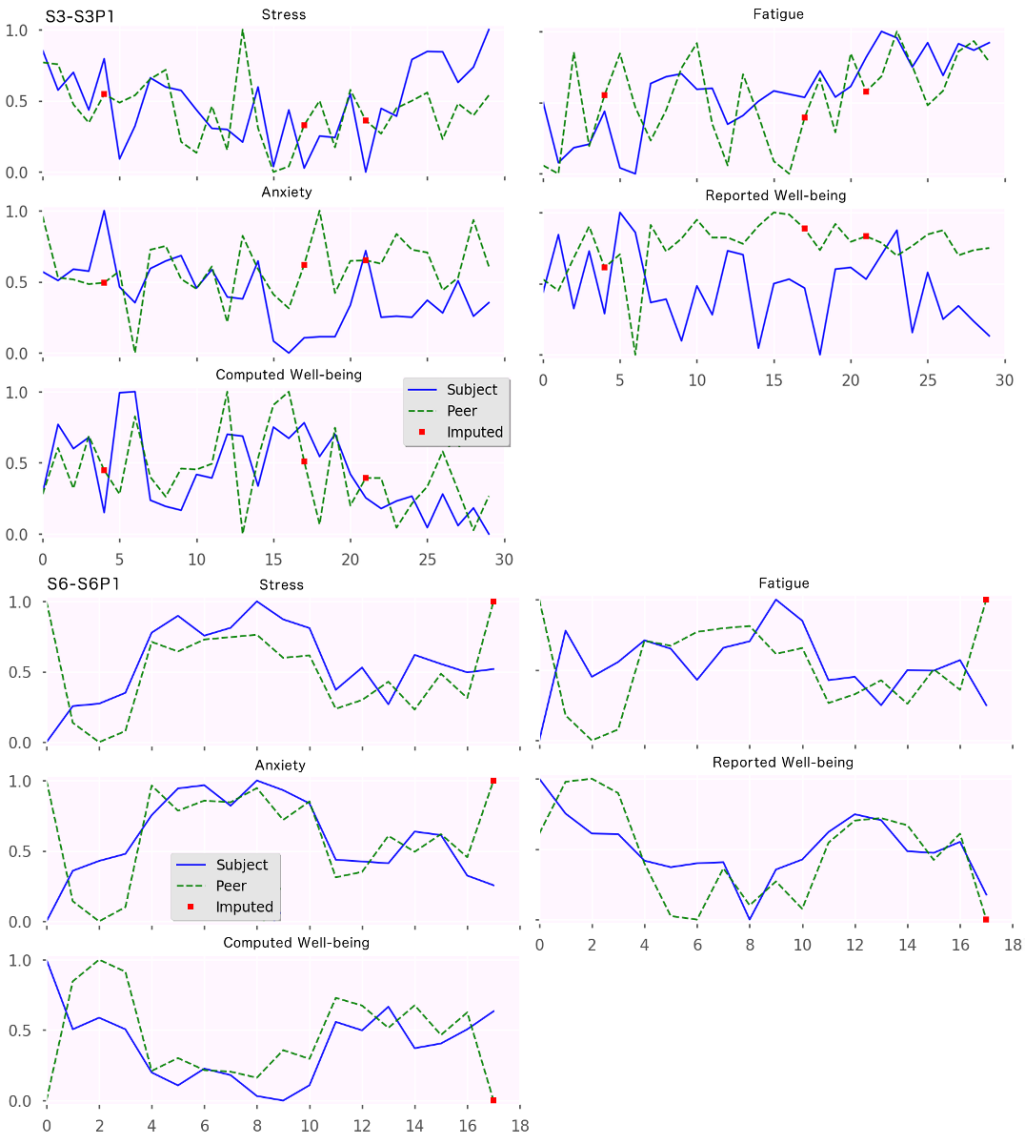
Ecological momentary assessment/peer-ceived momentary assessment. Plots from Study B. The x-axis represents days in the study, and the y-axis represents the magnitude of the normalized assessments for stress, fatigue, anxiety, and computed well-being.

Figure 3, similar to Figure 2, presents sample plots from *study B*. In the 5 plots on the top, corresponding to the dyad *<*S3-S3P1*>*, we observed high agreement for stress and fatigue and low agreement for anxiety. Additionally, in this particular case, the assessments for reported well-being differed significantly, whereas the computed well-being showed high agreement (in fact, their median was statistically equal). However, this could be because of either the participant or the peer answering the question of well-being on assigning higher importance to other aspects not inherently reflected as stress, fatigue, and anxiety (which are the sole variables used in our pragmatic computation of well-being, as defined in the *Methods* section).

Finally, the 5 plots at the bottom of Figure 3, for the dyad *<*S6, S6P1*>,* show very high agreement in all the states, and their median was statistically equal in all the cases. Assuming their observations genuinely reflect the actual state of the participant, with approximately 3 weeks of agreement in which there were high and low episodes, this dyad seems to have a shared understanding of the concepts and excellent sensing skills by the peer. Such a dyad raises the confidence of a researcher to include them in a longitudinal study in which periods of disagreement between EMA and PeerMA motivate the use of other qualitative methods (such as the Day Reconstruction Method [61]) to study the root causes of such disagreements.

#### Mean Directional Accuracy of EMA/PeerMA

Various techniques can be used to quantify the daily agreement between the EMAs and PeerMAs. For instance, residual analysis such as mean absolute percent error (MAPE) or more robust alternatives such as mean arctangent absolute percentage error (MAAPE) [62]. In our case, the robustness of MAAPE is derived from the fact that MAPE produces undefined values when the assessment of a participant is equal to 0. These techniques can quantify the overall difference between the EMA and the PeerMA values over time. However, for the particular application of PeerMA, we expect to see periods of disagreement as they represent the differences between the self-assessments and the observer assessments that are worth identifying and analyzing during field applications.

Therefore, we analyzed the daily agreement between the EMAs and the PeerMAs as follows. As a first approach, we reported the mean directional accuracy (MDA), which measures the agreement (ie, we report *a match*) in the direction of change of momentary assessments between participants and peers. MDA considers only the direction of the change (eg, upward or downward) and not its magnitude. For instance, let us suppose that a participant reported (*t*_0_*,* 0*.*4)*,* (*t*_1_*,* 0*.*3)*,* and (*t*_2_*,* 0*.*7) and a peer reported (*t*_0_*,* 0*.*2)*,* (*t*_1_*,* 0*.*4)*,* and (*t*_2_*,* 0*.*6); then, between *t*_0_ and *t*_1_, there is a mismatch as the participant’s report decreased (negative direction) whereas the peer’s report increased (positive direction). From *t*_1_ to *t*_2_, however, there is a match as both participants’ and peers’ reports increased (0.3 to 0.7 for the participant and 0.4 to 0.6 for the peer).

**Figure 4:**
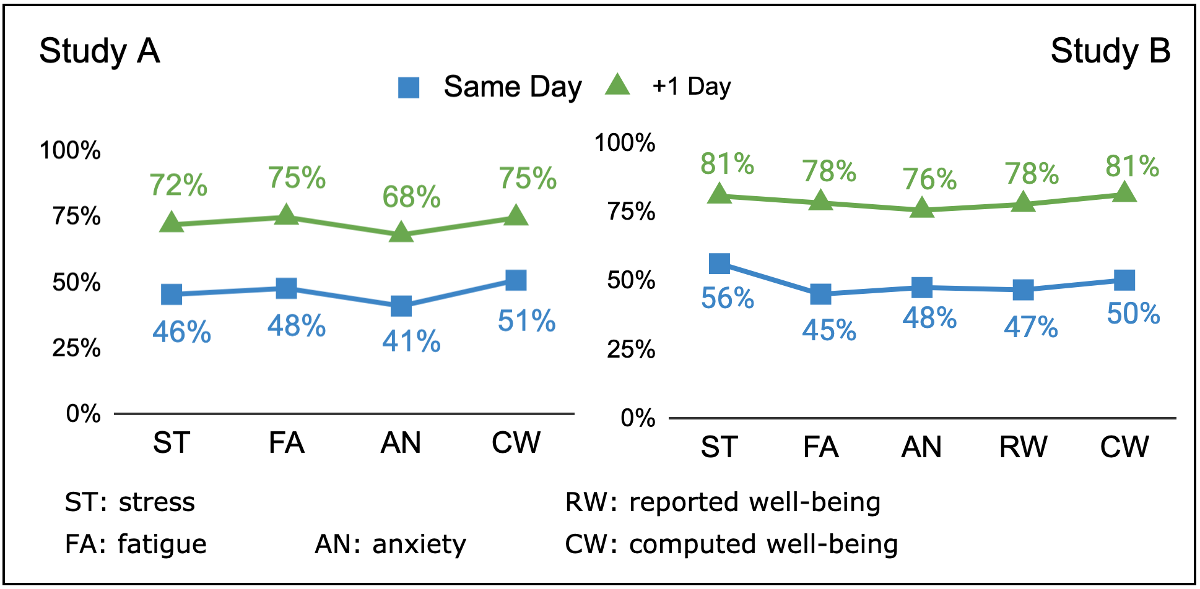
Mean Directional Accuracy. “Same Day" is the average percentage of days that participants and peers agreed in the directional change of their assessments the same day (close to chance). “+1 Day" is the average percentage of days that participants and peers agreed in the directional change of their assessments the same day or the day after.

In the 2 studies, the number of assessments between participants and peers differed every day. Hence it was not possible to calculate the MDA for each individual EMA/PeerMA. Thus, we calculated the daily average for stress, fatigue, anxiety, and well-being for each participant and peer using all the assessments given that day. We then counted the number of days with *matches* and divided it by the number of days for which both the participant and the peer issued at least one assessment. Figure 4 shows the accumulated MDA results for both studies. *Same day* is the percentage of days that participants and peers agreed in the directional change of their assessments the same day. As can be seen from the figure, the result is close to chance. However, it increased to approximately 73% in *study A* and to 79% in *study B* on counting matches occurring the same day or 1 day after. Naturally, MDA can be calculated with higher granularity down to every consecutive pair of EMA/PeerMA values. Overall, MDA shows that PeerMA is promising for identifying variations in mental or physical health early on (eg, accurately assessing changes in the individuals’ day-to-day states).

### EMA and PeerMA Correlations

#### EMA/PeerMA Spearman Correlations

To further investigate the values of EMA and PeerMA, we conducted a correlation analysis for EMA/PeerMA values in both studies. By focusing on the correlation, we did not assume that the EMA and the PeerMA measure the same constructs; we investigated it later in this paper. Therefore, in this study, we applied the Spearman rank correlation method because (1) participant and peer assessments are not independent, they both refer to a state of the participant and (2) the Shapiro-Wilk, D’Agostino *K*^2^, and Anderson-Darling tests indicate that not all individuals’ and peers’ assessments were normally distributed.

For *study A*, the Spearman rank correlation coefficients for each of the states are presented in Table 8. Each row presents the correlation coefficient *r_s_* and *P* value for a participant-peer dyad. We also show the correlation between the computed well-being of each participant and peer. In Table 9, we present a summary of the values from Table 8 classified in 6 correlation groups. In this sample, 40% of the correlations are weakly positive, followed by 36% being weakly negative and 20% being moderately positive.

**Table 8:** Study A: ecological momentary assessment/peer-ceived momentary assessment Spearman correlations calculated throughout the study. Each row shows the correlation between the participants’ and peers’ assessments.

Participant ID	Peer ID	Stress		Fatigue		Anxiety		Computed well-being		
		r_s_	*P* value	r_s_	*P* value	r_s_	*P* value		r_s_	*P* value
S1	S1P1	0.44	.02	0.28	.15	0.57	.002		0.58	.001
S2	S2P1	0.50	.003	0.26	.15	0.24	.17		0.63	<.001
S3	S3P1	–0.18	.36	–0.16	.40	–0.09	.64		–0.15	.45
S4	S4P1	0.06	.76	–0.10	.62	–0.09	.64		0.09	.67
S5	S5P1	–0.10	.62	–0.23	.24	–0.35	.07		–0.19	.33
S6	S6P1	–0.23	.23	–0.11	.57	0.27	.16		–0.08	.66
S7	S7P1	0.19	.33	–0.01	.96	–0.31	.11		–0.13	.49
S7	S7P2	–0.01	.96	0.28	.14	0.19	.32		0.36	.05
S8	S8P1	0.11	.57	0.08	.68	0.22	.25		0.26	.18
S8	S8P2	0.32	.10	0.59	<.001	0.33	.08		0.63	<.001
S9	S9P1	–0.25	.16	–0.05	.80	–0.28	.10		–0.37	.03
S9	S9P2	0.02	.91	0.29	.10	–0.09	.60		–0.16	.38
S10	S10P1	0.29	.13	–0.18	.35	0.04	.84		0.35	.06
S10	S10P2	0.14	.48	0.05	.80	–0.15	.45		–0.14	.46
S11	S11P1	0.39	.03	0.39	.03	–0.06	.75		0.28	.13
S11	S11P2	0.07	.70	0.03	.89	–0.06	.75		0.15	.42
S12	S12P1	0.32	.10	0.27	.16	0.21	.28		0.56	.002
S12	S12P2	0.07	.73	–0.24	.22	–0.02	.94		0.07	.71
S13	S13P1	0.41	.02	–0.19	.30	0.41	.02		0.27	.14
S13	S13P2	0.36	.05	–0.34	.06	0.43	.02		0.27	.15

**Table 9:** Study A: summary of ecological momentary assessment/peer-ceived momentary assessment Spearman correlations.

Correlation strength	Stress, n (%)	Fatigue, n (%)	Anxiety, n (%)	Computed well-being, n (%)	Total, n (%)
Highly positive^a^	0 (0)	0 (0)	0 (0)	0 (0)	0 (0)
Moderately positive^b^	5 (25)	2 (10)	3 (15)	6 (30)	16 (20)
Weakly positive^c^	10 (50)	8 (40)	7 (35)	7 (35)	32 (40)
Weakly negative^d^	5 (25)	9 (45)	9 (45)	6 (30)	29 (36)
Moderately negative^e^	0 (0)	1 (5)	1 (5)	1 (5)	3 (4)
Highly negative^f^	0 (0)	0 (0)	0 (0)	0 (0)	0 (0)

^a^(0.67 to 1.00): Values of the spearman correlation inside this interval are considered highly positive.

^b^(0.34 to 0.66): Values of the spearman correlation inside this interval are considered moderately positive.

^c^(0.00 to 0.33): Values of the spearman correlation inside this interval are considered weakly positive.

^d^(−0.33 to 0.00): Values of the spearman correlation inside this interval are considered weakly negative.

^e^(−0.66 to −0.34): Values of the spearman correlation inside this interval are considered moderately negative.

^f^(−1.00 to −0.67): Values of the spearman correlation inside this interval are considered highly negative.

For *study B*, the Spearman rank correlation coefficients for each of the states are presented in Table 10. In this case, we reported the correlations between the *reported well-being* of each participant and peer. Correspondingly, Table 11 summarizes the values from Table 10. In this study, 43% of the correlations were weakly positive, followed by a tie of 23% between weakly negative and moderately positive.

**Table 10:** Study B: ecological momentary assessment/peer-ceived momentary assessment Spearman correlations calculated throughout the study. Each row shows the correlation between the participants’ and peer’s assessments calculated throughout the study.

Participant ID	Peer ID	Stress		Fatigue		Anxiety		Reported well-being			Computed well-being	
		r_s_	*P* value	r_s_	*P* value	r_s_	*P* value	r_s_	*P* value	r_s_		*P* value
S1	S1P1	–0.39	.02	–0.36	.03	–0.33	.05	0.18	.30	–0.30		.08
S2	S2P1	–0.31	.10	0.33	.08	0.26	.18	0.40	.03	0.13		.51
S3	S3P1	0.37	.04	0.44	.01	0.04	.84	–0.08	.68	0.44		.01
S4	S4P1	0.24	.21	0.16	.42	0.10	.60	–0.17	.38	0.37		.048
S5	S5P1	0.04	.84	0.03	.88	0.06	.76	0.28	.13	–0.01		.95
S6	S6P1	0.44	.07	–0.06	.81	0.24	.34	0.83	<.001	0.26		.30
S7	S7P1	0.41	.03	0.20	.29	–0.25	.19	0.58	<.001	–0.58		.001

**Table 11:** Study B: summary of ecological momentary assessment/peer-ceived momentary assessment Spearman correlations.

Correlation strength	Stress	Fatigue	Anxiety	Reported well-being	Computed well-being	Total, n (%)
Highly positive^a^	0 (0)	0 (0)	0 (0)	1 (14)	0 (0)	1 (3)
Moderately positive^b^	3 (43)	1 (14)	0 (0)	2 (29)	2 (29)	8 (23)
Weakly positive^c^	2 (29)	4 (57)	5 (71%)	2 (29)	2 (29)	15 (43)
Weakly negative^d^	1 (14)	1 (14)	2 (29%)	2 (29)	2 (29)	8 (23)
Moderately negative^e^	1 (14)	1 (14)	0 (0)	0 (0)	1 (14)	3 (9)
Highly negative^f^	0 (0)	0 (0)	0 (0)	0 (0)	0 (0)	0 (0)

^a^(0.67 to 1.00): Values of the spearman correlation inside this interval are considered highly positive.

^b^(0.34 to 0.66): Values of the spearman correlation inside this interval are considered moderately positive.

^c^(0.00 to 0.33): Values of the spearman correlation inside this interval are considered weakly positive.

^d^(−0.33 to 0.00): Values of the spearman correlation inside this interval are considered weakly negative.

^e^(−0.66 to −0.34): Values of the spearman correlation inside this interval are considered moderately negative.

^f^(−1.00 to −0.67): Values of the spearman correlation inside this interval are considered highly negative.

#### EMA/PeerMA Subcorrelations Including Entry Survey Results

We conducted 3 more correlation analyses relevant to these studies, including the entry survey reports (GERT, PSS, and SDS) as collected within the studies. We again chose the Spearman ranked correlation method because of the small number of samples—17 for *study A* (after removing 3 participant-peer pairs who did not complete the entry surveys) and 7 for *study B*. This small sample size did not permit an accurate assessment of the underlying distribution of the data; hence, we did not assume that the samples were normally distributed. We report results relevant for the feasibility study conducted, even when they were not statistically significant.

#### EMA/PeerMA Values Versus Entry Surveys

The correlation between participants’ and peers’ SDS, PSS, and self-considered stressed score (from Table 4), and the *r_s_* coefficient from the Spearman rank correlation for each state (from Table 8 and Table 10) was assessed. This was important to identify direct relationships between individual characteristics (obtained in the entry surveys) and the observed agreement between participants and peers. In *study A*, we found that the higher the participants’ SDS scores, the lower the *r_s_* correlation coefficient for fatigue (Table 8; *r*=*−*0*.*52; *P*=*.*03). The result also holds true for *study B*, although it was not statistically significant (Table 10; *r*=*−*0*.*40; *P*=*.*38). Moreover, the more stressed the peers considered themselves (*r*=0*.*45; *P*=*.*07), the higher the correlation was between EMA/PeerMA and anxiety (Table 8). The result also holds true in *study B* (Table 10; *r*=0*.*82; *P*=*.*02).

#### Medians of Within-EMA/PeerMA Value

For both participants and peers, we derived the correlation between the median of the following pairs of states: stress-fatigue, stress-anxiety, stress–computed well-being, fatigue-anxiety, fatigue–computed well-being, and anxiety–computed well-being. *Study B* included the combinations with reported well-being in addition to the aforementioned pairs of states. This is relevant to understand whether participants and peers treat and report some of the states alike (ie, with the same semantics). We found no correlation between stress-fatigue or fatigue-anxiety for neither participants nor peers in both studies. Nevertheless, we observed a positive and close to statistically significant correlation in stress-anxiety for participants and peers in both studies. In *study A*, for participants: *r*=0*.*73*; P*=*.*001 and for peers: *r*=0*.*44*; P*=*.*08. In *study B*, for participants: *r*=0*.*64*; P*=*.*12 and for peers: *r*=0*.*95*; P*=*.*001. Moreover, in *study B*, we did not observe a consistent or statistically significant correlation between reported and computed well-being for participants (*r*=0*.*36; *P*=*.*42) or peers (*r*=*−*0*.*39; *P*=*.*38). This result suggests that participants and peers may have considered other important variables beyond the assessments of stress, fatigue, and anxiety when they answered the question about well-being.

#### Median of EMA/PeerMA Values

The correlation between the participants’ median of stress/fatigue/anxiety/well-being and the peers’ median of the perceived state was assessed. This is important to understand whether there is high or low agreement in the assessments of the states at the sample level. We used the median because, at the individual level, the assessments are not independent and are not normally distributed. In *study A*, we observed a negative correlation for stress (*r*=*−*0*.*39; *P*=*.*11), a positive correlation for fatigue (*r*=0*.*36; *P*=*.*15), and a weakly negative correlation for computed well-being (*r*=*−*0*.*14; *P*=*.*58), although none of them was statistically significant. In *study B*, we observed a positive correlation for stress (*r*=0*.*54; *P*=*.*20), anxiety (*r*=0*.*52; *P*=*.*22), and computed well-being (*r*=0*.*39; *P*=*.*38), although, again, none of them was statistically significant.

### EMA/PeerMA: Statistical Agreement

After the correlations evaluated within the previous sections, we focus on the statistical agreement of the EMA/PeerMA assessments by which we assume that the EMA and PeerMA measure the same constructs. To quantify the overall agreement between EMA and PeerMA, we applied the Wilcoxon signed-ranked test to determine whether the medians of the 2 sets (EMAs from participants and PeerMAs from peers) are statistically equal. The justification for choosing this test is that (1) participants’ and peers’ assessments are not independent; (2) the participant and peers’ assessments are paired; and (3) in our datasets, not all individual and peer assessments are normally distributed. The null hypothesis, *H*_0_, of the Wilcoxon signed-ranked test is that the 2 samples have the same distribution. Thus, failing to reject *H*_0_ suggests that participants’ and peers’ assessments are statistically equal.

For *study A*, the dyads *<*participant, peer*>* for which at least one of the states is statistically equal (ie, *P>*.05) are presented in Table 12. For this particular study, 40% (8/20) of the peers reported daily stress assessments, which are statistically equal to those reported by the participant. The values obtained were 30% (6/20) for fatigue, 55% (11/20) for anxiety, and 35% (7/20) for computed well-being.

The results for *study B* are presented in Table 13. In this case, 29% (2/7) of peers reported daily stress assessments, which are statistically equal to those reported by the participant. The values were 43% (3/7) for fatigue and anxiety and 71% (5/7) for both reported and computed well-being.

**Table 12:** Study A: Wilcoxon signed-ranked significance tests for ecological momentary assessment/peer-ceived momentary assessment (*P*=.05).

Peer ID	*P* value (stress)	*P* value (fatigue)	*P* value (anxiety)	*P* value (computed well-being)
S1P1	.001	<.001	<.001	.34
S2P1	<.001	<.001	.58	.002
S3P1	.74	.004	.21	<.001
S4P1	<.001	.19	.07	.002
S5P1	<.001	.03	.09	<.001
S6P1	.96	.63	.09	.61
S7P2	.003	.002	.001	.18
S8P2	<.001	.13	<.001	<.001
S9P1	<.001	.02	<.001	.23
S9P2	<.001	.05	.001	<.001
S10P1	<.001	<.001	.02	.28
S10P2	.36	.01	.29	.72
S11P1	.37	.003	.40	<.001
S11P2	.77	<.001	.10	<.001
S12P1	.22	.01	<.001	<.001
S12P2	.03	.82	.94	<.001
S13P1	.06	<.001	.12	.002
S13P2	.13	.43	.80	.19

**Table 13:** Study B: Wilcoxon signed-ranked significance tests for ecological momentary assessment/peer-ceived momentary assessment (*P*=.05).

Peer ID	*P* value (stress)	*P* value (fatigue)	*P* value (anxiety)	*P* value (reported well-being)	*P* value (computed well-being)
S1P1	.003	.03	.01	.07	.42
S2P1	.001	.02	.002	.30	<.001
S3P1	.21	.18	.01	<.001	.50
S4P1	.01	.29	.07	.64	.05
S5P1	.02	.02	.35	.03	.13
S6P1	.06	.50	.56	.45	.06
S7P1	<.001	.03	.01	.07	.01

### Operational and Human Factors

#### Users’ Reflections on the mQoL Peers App

##### Positive Aspects

In general, users found the app easy to use and liked the brief surveys. Some users said that the app helped them to be more aware of their emotions. We quote some of their comments, *study A*: (subject) “Thanks to it I tried to be more aware of my stress level during the day” and (subject) “It makes me think about my attitude and feelings at the moment.“ For *study B*: (subject) “I liked that surveys were short and easy to understand” and (subject) “I liked the use of scale and colors I wish I can see the results.“ The users acknowledged the minimal obtrusiveness we aimed for.

##### Negative Aspects

For *study A*: (subject) “App is not very fun, the exercise quickly becomes monotonous” and (peer) “Same 4 questions 1 month extremely repetitive and annoying.“ For *study B*: (subject) “The app needs more questions and a dashboard” and (peer) “Short questions could be together in one page.“ The users acknowledged what we already knew, that the study length and frequency of self-reports are important for the quality of the data collected. In the *Discussion* section, we present implications stemming from these observations as well as future work areas related to the findings.

#### Users’ Reflections on the Studies

##### Participants’ Reflections

We asked participants how they felt about reflecting on their own emotional states. For *study A*, some participants confirmed a known risk of reactivity in *EMA*: (subject) “Sometimes the app has accentuated my stress” or (subject) “Answering the anxiety question often made me feel anxious.“ Other participants reported enriching experiences:

I had the impression of being more aware of my anxiety during the study, before, I did not pay special attention to it.

Subject

*It made me take a step back on myself. For example, when I was stressed, I told myself that I had to calm down. When I had high fatigue, I said to myself that I had to sleep better to recover, not look at my phone before sleeping.*

Subject

For *study B*, the experiences were mostly positive, for example:

It allowed me to anchor myself and see what is causing me to feel stressed.

Subject

Loved it. It made me more aware of my emotional states than ever before.

Subject

The higher sampling frequency in *study A* (8 per day vs 3 per day in *study B*) could explain the unintended side effects experienced by some of its participants.

##### Peers’ Reflections

We asked peers how they felt about reflecting on someone else’s emotional states during the study. In both studies, some peers said that the task was challenging to complete at times. Others said that participating in the study allowed them to learn more about emotional states. For example, for *study A*: (peer) “Sometimes it was hard because we hadn’t talked for hours” and (peer) “If I did not see her, I had no idea what her emotional state was.“ On the contrary, peers reporting more enriching experiences said:

I have the feeling to take stress problems more seriously, not like everyone is stressed and it is normal, but to understand that stress can block life of some people.

Peer

It made me think if she is doing well and this experience made me write to her more often.

Peer

For *study B* these peers stated:

It was a little harder than I had expected. I typically use facial expression and tone to determine my friends emotional states. On days that I don’t see her, I’d have to rely on how she texts.

Peer

It allowed me to learn about his mood every day and know him better and any problems going on.

Peer

### Implications of Technology Choices

This section summarizes the results related to the technology choices in the 2 studies and how they may influence the methods’ feasibility. In the 2 studies, we assumed that both EMA and PeerMA have the same technological requirements: (1) the mechanism to trigger the questions to participants at desired moments and (2) the channel to trigger the questions to the user and collect the answers reliably. As explained in the *Tools* section, we implemented EMA and PeerMA using our own *mQoL Lab* platform, detailed in the study by Berrocal et al [51].

To trigger the questions, van Berkel et al [63] provide a comprehensive overview of the multiple options researchers can choose from. We used uniformly randomized signal-contingent triggers throughout the day (waking hours). In *study A*, the *mQoL Peers* app was responsible for scheduling and triggering the signals in the participants’ smartphones to present an EMA. These signals triggered a push notification to the peers’ smartphones to initiate a corresponding PeerMA. This approach had 2 disadvantages: (1) it used computing resources of the users’ smartphones and (2) the users could (accidentally or intentionally) change the system settings causing negative consequences for the study. In *study B*, we reduced those risks and obtained favorable results by scheduling and triggering the signals from a server component, also via push notifications, simultaneously to both participants and peers.

Regarding the channel itself, smartphones are commonly used for mobile human studies as they are often close to the owner [64]. However, cross-platform compatibility may be an obstacle as the smartphone type would need to be used as inclusion criteria, potentially biasing the study results. We had this experience ourselves. Namely, for *study A*, our platform was compatible only with Android. For *study B*, we improved our platform to support iOS-based devices either by the participant, the peer, or both. Nevertheless, in *study B*, 2 senior candidates were excluded during the study recruitment phase as they did not use the smartphone often and their preferred channel was their tablet computer. Researchers designing studies may consider this audience as well and design their study accordingly.

### Suggestions From Users About Technology and Methods

The following are recommendations made by users (participants and peers) in our studies. First, they wanted to have some kind of dashboard to see their previous assessments and track how many they completed each day. This may have positive effects on response compliance; however, it may imply higher reactivity to the study itself, where a momentary, ecologically valid EMA may be influenced by the number or content of the past EMAs (depending on the dashboard design). The participants also wanted greater freedom to select the moods or states that they felt confident about reporting at a given moment. Such a level of freedom is possible, but it should be done carefully to salvage the collection of ecologically valid EMAs and PeerMAs, assuring the joint understanding of participants/peers of the state being assessed (eg, snacking on foods throughout a day) as well as to ensure that the data being collected directly relates to the main goal of the study. Additionally, such a participant-driven EMA/PeerMA study design may result in bias stemming from collecting only the states the participants want to report.

One peer in *study A* (whose relationship with the subject was “a friend from the university”) suggested that the participants’ assessment should start with a question: “Is your peer next to you to answer a short survey?“ This is a valid observation that can be explored in future studies as a way to study the validity of the PeerMA answers. In our studies, peers were able to express their confidence levels for each individual PeerMA. They may have selected low confidence if they had not been in contact (either physical or virtual) with the participant in the recent past. Future research could include software assessment of proximity between participants and peers (eg, using Bluetooth, using Wi-Fi, using Geo Fences, or leveraging the social network apps’ usage patterns) as a way to reduce the burden for peers being requested to assess the state of the individual when the probability of obtaining a reliable assessment is low.

## Discussion

### Structure

In summary, our first research aim was to explore the feasibility of applying the PeerMA method to involve peers to assess phenomena such as mental states in healthy individuals. The second aim was to determine operational aspects and human factors that need to be taken into account to most effectively use the PeerMA method. We reflect upon the overall results to answer these questions.

### Feasibility of Using PeerMA

In this section, to address the first research aim, we summarize the experience from both studies related to the tangible contributions from participants and peers using both the EMA and the PeerMA methods.

Regarding the users’ recruitment and participation, we conclude the following: we first noticed that some participants had difficulties finding a peer. In *study A*, we initially had 18 participants; 5 participants who attempted to enroll and agreed to try and find a peer stopped later, indicating that they were unable to find a peer to participate. Hence, we ended up with 13 participants with peers in *study A*. In *study B*, we also started with 18 participants, with 8 cases in which the participant enrolled in the study but never enrolled a peer. Two of them informed us about the situation and six stopped without further contact. The three participants who completed the full study but did not manage to involve a peer were excluded from the analysis.

Once enrolled, participant retention was high. Although recruiting participants with peers required more effort than recruiting participants for EMA-only studies, we successfully completed two field studies of 4 weeks’ duration in two geographically distant locations, as described here. In both studies, we observed that almost all participants who enrolled with a peer from the beginning were able to continue in the study till the end, on average, 29 (SD 1*.*7) days for participants and 25 (SD 5*.*9) days for peers in *study A* and 27 (SD 4*.*5) days for participants and 27 (SD 4*.*8) days for peers in study B. However, we noticed that some individuals had a low EMA/PeerMA response rate, as shown in Table 5, especially in *study A* (<10%). We believe this was at least partially due to a high number of daily EMA/PeerMA notifications (8 per day) that came along usual participants and peers’ obligations during the day. We limited the number of notifications in *study B* to 3 per day, which resulted in a better EMA/PeerMAs response rate (Table 5; only 1 peer had a response rate as low as 30%).

When it comes to the overall agreement between EMA and PeerMA assessments, the evidence for the feasibility of the PeerMA method is as follows. Although not conclusive or generalizable, we observed a strong, close to statistically significant correlation between participant and peer’s assessments of stress and anxiety (for *study A*, participants: *r*=0*.*73*; P*=*.*001 and peers: *r*=0*.*44*; P*=*.*08 and for *study B*, participants *r*=0*.*64*; P*=*.*12 and peers *r*=*.*95*; P*=*.*001; details in the *EMA and PeerMA Correlations* section). In both studies, the more stressed the peers described themselves in the entry survey (*study A*: *r*=0*.*45*; P*=*.*07 and *study B*: *r*=0*.*82*; P*=*.*02), the higher the correlation between EMA and PeerMA for anxiety (Table 8 and Table 10, respectively; details in the *EMA and PeerMA Correlations* section). Collectively, this suggests that stressed individuals tend to be more effective in detecting anxiety in their peers (already studied by Harrigan et al [65]) and that anxiety could be a proxy for the level of stress of a person as stress and anxiety were positively correlated in these particular samples.

Additionally, we recall the overall percentage of peers who reported daily assessments statistically equal to those reported by their participants. In *study A*, the results were 40% for stress, 30% for fatigue, 55% for anxiety, and 35% for computed well-being. In *study B*, the results were 29% for stress, 43% for fatigue and anxiety, and 71% for computed and reported well-being (details in the *EMA and PeerMA Statistical Agreement* section). In summary, in both studies, participants and peers achieved higher agreement in their assessments of anxiety and fatigue and lower agreement in their assessment of stress. We reason for this result as follows.

Anxiety is a complex state and highly involuntary [65], and it is presumably difficult to hide it as specific individuals try to do with stress. Manifestations of anxiety are anchored to a particular event or situation. Close peers who are aware of the events or situations can use that knowledge to estimate the state of the person [58]. Alternatively, a high agreement could also result from a case in which participants and peers interpret stress and anxiety as the same or very similar state and report it accordingly.

In addition, we consider that detecting fatigue in peers is less challenging as people tend to talk about it openly. Nevertheless, one person may be highly fatigued after 1 night of poor sleep, whereas another person may not reach that same level after 2 or 3 nights of poor sleep. For this purpose, psychometric models at the individual level help make comparisons among the assessments [58].

Finally, stress has a social component affected by stereotypes [66] (eg, work or school performance, fear of public speaking), and the social (external) manifestation of stress may produce different physiological (internal) reactions and external observable behaviors among individuals. It is ultimately the physiological stress that causes the underlying threat to a person’s health. Potentially due to the social complexity of the stress phenomena, our observed results do not lead to strong conclusions for employing PeerMA when assessing stress. Nevertheless, the combined use of EMA and PeerMA within a specific study, to complement other data collection methods (eg, psychophysiology), represents a possible way to study peer-based stress assessment further.

In summary, there are challenges and open questions regarding interoceptive awareness (ability to consciously sense the inner state of the body) to consider when using EMA to study emotional and physical states [67]. PeerMA is constrained by similar, but not identical, limitations as peers report about a state that occurs in the individual being observed [58]. Nevertheless, a frequent and careful pairing of self-assessments and others’ assessments (as shown in Figures 2 and 3), although subjective, may lead to new sources of information that we consider valuable to study how a person’s emotional and physical states unfolded over time. The results of our studies suggest that applying the PeerMA method to the study of complex phenomena, such as mental states in individuals, is feasible and can open up new perspectives to examine the relationships between self-assessments and others’ perceptions that are not possible to obtain from studies based on surveys or EMAs alone.

### Human Factors and Operational Aspects of PeerMA

We address the second research aim by summarizing operational aspects and human factors derived from the experience of using the PeerMA method in the two studies. We discuss the implications of certain technological choices and offer recommendations for researchers who wish to include the method in their studies.

#### Recruiting and Retaining Participants

To begin with, recruiting participants for studies is a known challenge [68]. Hence, we know that recruiting participants for paired studies that combine PeerMA with EMA is challenging as well. In both studies, we observed that several participants who initially applied to join the study did not actually enroll after the web-based meeting with the researcher (in which they learned that bringing a peer was a requirement for these studies). In *study A*, 5 participants who actually enrolled and agreed to try and find a peer later stopped participating, indicating that they were unable to find a peer willing to participate. In *study B*, we observed 8 cases in which the participant enrolled in the study but never enrolled a peer. Two of them informed us about the situation, whereas the remaining six simply stopped without further contact.

We found that one recommendation is to start applying the PeerMA method in cohorts for which reaching out to a peer becomes less complicated, and more motivating and valuable for both the participants and the peers. For instance, at the time of this writing, we are conducting one study with adult patients of the Stanford Medical Center recovering from a liver transplant. In this case, the patients answer EMAs, whereas their support person answers PeerMAs. Recruiting peers for this particular group was more straightforward because of the anticipated clinical value of such a study.

Another recommendation, which relates to the technological choices influencing the feasibility of the methods, is to include gamification techniques (eg, scoring points, winning prizes, and solving puzzles) as part of the study dynamics, which can provide an incentive for users to contribute data along the study duration and complete the study [69,70].

#### PeerMA: Methodological Opportunities

On the contrary, based on the experience during these two exploratory studies, we found it worth exploring how the EMA and PeerMA can be combined during a study to provide accurate, timely information about the observed participant state (and its changes). One suggestion is to modulate the administration of EMA and PeerMA based on prior knowledge about the participants’ state of interest and individual just-in-time answers. This would imitate the computerized adaptive tests, in which the questions are tailored to the past answers of the individual. In our case, the question of well-being (or a similarly discriminating question) could always be first. From our study design and results, we know that well-being encompasses stress, fatigue, or anxiety, and potentially, other states. Then, the next question would be chosen based on the answer (high or low well-being); for low well-being, relevant question(s) for the current state (eg, stress, fatigue, or anxiety) would be triggered to the participant and the peer. For high well-being, the survey may be completed. This approach may reduce the participants’ burden of monotonously answering questions when there may not be new information to provide. In other cases, the peers could be asked only to validate the response of the participant (eg, “Have you taken your medication?”). Nevertheless, some participants may feel their privacy is at stake when their peers answer a PeerMA every time they answer an EMA. Participants could have the option to decide—in real time—not to send the PeerMA to peers if they want to regain control over their privacy.

Another variation for the PeerMA design is to give users the options to slightly customize the time windows when they feel available and willing to answer certain types of questions. Some of them may be more engaged if they can choose the type of signal as well as the time window when they are better prepared to take an EMA or PeerMA (in a similar way as they make other daily choices like taking a cup of coffee or making a personal phone call). On the other hand, this could be detrimental to the research if participants are preparing to give a specific answer, knowing that the questions are being triggered at specific times, or if they choose to not answer questions at times when they feel more stressed, causing data loss and result bias. Additionally, some studies with PeerMA may allow users to take both the roles of participants and peers simultaneously, which allows them to report on each other’s states, which may increase engagement.

Overall, as an exploratory method, there are yet many opportunities to design studies leveraging PeerMA. The main methodological question relates to what information or measure about the user state could be collected in reliable and minimally obtrusive ways from the participant and his/her peers. If chosen properly, we believe that such information collected from participants and peers simultaneously could enable further understanding of the observed state.

### Limitations and Future Work

One limitation of these studies is the lack of ground truth of the assessed conditions (stress, fatigue, or anxiety). Despite the data presented in this preliminary analysis, we were unable to determine whether the EMA or the PeerMA were closer to the actual state of the participants. The limitation is inherent to any self-report, EMA-based study. To further examine the reliability of PeerMA, more research is needed to incorporate more modalities, such as heart rate variability for physiological stress.

Another limitation of our work is the small sample size of participants and peers. As indicated earlier, a larger sample is necessary to further investigate the reliability of the assessments as well as the effects introduced by sample characteristics such as the amount of time peers interact with the participants during a day, type of relationship between participant and peer, or coresidence as reported by Neumann et al [8], among others.

Additionally, research is needed to examine whether PeerMA affects the usual behavior of the participant during a study. Namely, if the participant, knowing that he/she is being observed, explicitly changes his/her state and behavior when interacting with the peer. Similarly, further research is needed to include the state of the peer at the moment of answering a PeerMA, for example, to understand the possible effects or biases related to the ego depletion theory [71].

Finally, our future work includes a case by case examination of how peers’ reported level of confidence (ie, low, moderate, or high), as well as other socioeconomic characteristics, influence the results derived from the EMA and PeerMA agreement.

### Conclusions

We presented results from two user studies conducted in the participants’ natural daily life environments, evaluating the first version of a platform implementing the PeerMA method deployed on users’ personal smartphones. The studies showed encouraging results from a total of 20 participants and 27 peers contributing multiple daily assessments for approximately 4 weeks each. In the studies, we collected empirical evidence regarding the feasibility of the method. We discussed the methodological and human aspects related to the application of the PeerMA method to study real-life phenomena, including those related to mental health. We demonstrated that users accepted the method and provided valuable feedback. We identified and discussed improvement opportunities that could lead to higher user engagement as well as more elaborate methodological options for researchers to explore when leveraging PeerMA in their studies. We discussed technical aspects to consider for a reliable, technology agnostic, and minimally obtrusive implementation of the PeerMA method.

We believe that the PeerMA method evaluated in this study opens a new perspective to study an individual’s state based on frequent and possibly paired observations from trusted peers beyond the information traditionally obtained with EMA. As an independent observation, it has value for applications in clinical settings to evaluate the severity of and support treatment of mental disorders such as OCD or addictions. However, more research is needed to guarantee reliable utilization with sufficient control to manage potential emergent biases stemming from either the participants or the peers or the momentary context in which PeerMA is triggered.

## Abbreviations

EMA: ecological momentary assessment
ESM: Experience Sampling Method
GERT: Geneva Emotion Recognition Test
MAAPE: mean arctangent absolute percentage error
MAPE: mean absolute percent error
MDA: mean directional accuracy
OCD: obsessive-compulsive disorder
PeerMA: peer-ceived momentary assessment
PSS: Perceived Stress Scale
SDS: Social Desirability Scale
UNIGE: University of Geneva
